# Selective Use of Sequential Digital Dermoscopy Imaging Allows a Cost Reduction in the Melanoma Detection Process: A Belgian Study of Patients with a Single or a Small Number of Atypical Nevi

**DOI:** 10.1371/journal.pone.0109339

**Published:** 2014-10-14

**Authors:** Isabelle Tromme, Brecht Devleesschauwer, Philippe Beutels, Pauline Richez, Nicolas Praet, Laurine Sacré, Liliane Marot, Pascal Van Eeckhout, Ivan Theate, Jean-François Baurain, Julien Lambert, Catherine Legrand, Luc Thomas, Niko Speybroeck

**Affiliations:** 1 Department of Dermatology, Centre du Cancer, Cliniques Universitaires St Luc, Université catholique de Louvain, Brussels, Belgium; 2 Institute of Health and Society, Faculty of Public Health, Université catholique de Louvain, Brussels, Belgium; 3 Centre for Health Economics Research & Modelling Infectious Diseases, Vaccine & Infectious Disease Institute, Faculty of Medicine & Health Sciences, University of Antwerp, Antwerp, Belgium; 4 Department of Biomedical Sciences, Institute of Tropical Medicine, Antwerp, Belgium; 5 Department of Medical Oncology, Centre du Cancer, Cliniques Universitaires St Luc, Université catholique de Louvain, Brussels, Belgium; 6 Department of Dermatology, Universitair Ziekenhuis, Antwerp, Belgium; 7 Institute of Statistics, Biostatistics and Actuarial Sciences, Université catholique de Louvain, Louvain-la-Neuve, Belgium; 8 Department of Dermatology, Lyon 1 University, Centre Hospitalier Lyon Sud, Pierre Bénite, France; University of Queensland Diamantina Institute, Australia

## Abstract

**Background:**

Dermoscopy is a technique which improves melanoma detection. Optical dermoscopy uses a handheld optical device to observe the skin lesions without recording the images. Sequential digital dermoscopy imaging (SDDI) allows storage of the pictures and their comparison over time. Few studies have compared optical dermoscopy and SDDI from an economic perspective.

**Objective:**

The present observational study focused on patients with one-to-three atypical melanocytic lesions, i.e. lesions considered as suspicious by optical dermoscopy. It aimed to calculate the “extra-costs” related to the process of melanoma detection. These extra-costs were defined as the costs of excision and pathology of benign lesions and/or the costs of follow-up by SDDI. The objective was to compare these extra-costs when using optical dermoscopy exclusively versus optical dermoscopy with selective use of SDDI.

**Methods:**

In a first group of patients, dermatologists were adequately trained in optical dermoscopy but worked without access to SDDI. They excised all suspicious lesions to rule out melanoma. In a second group, the dermatologists were trained in optical and digital dermoscopy. They had the opportunity of choosing between immediate excision or follow-up by SDDI (with delayed excision if significant change was observed). The comparison of extra-costs in both groups was made possible by a decision tree model and by the division of the extra-costs by the number of melanomas diagnosed in each group. Belgian official tariffs and charges were used.

**Results:**

The extra-costs in the first and in the second group were respectively €1,613 and €1,052 per melanoma excised. The difference was statistically significant.

**Conclusions:**

Using the Belgian official tariffs and charges, we demonstrated that the selective use of SDDI for patients with one-to-three atypical melanocytic lesions resulted in a significant cost reduction.

## Introduction

Cutaneous melanoma is one of the prime causes of death by cancer in the young Caucasian adult population [1]. Incidence, expressed as lifetime risk, is around 1–2% in Western Europe and the US and is still increasing in many countries [2]. Early detection and immediate surgery is the most effective treatment in reducing mortality. In order to favor this early detection, a technique called “dermoscopy” has been introduced in 1980s.

Optical dermoscopy (OD) is a non-invasive technique that uses a handheld, magnifying, optical device that suppresses light reflection by the stratum corneum either by liquid immersion or by cross-light polarization. It allows the observation of features invisible to the naked eye. Its efficacy to improve diagnostic accuracy for melanoma in a clinical setting has been proven in a meta-analysis [3]. However, this improvement is linked to examiners’ training and experience in the technique [4].

Sequential digital dermoscopy imaging (SDDI) allows the storage and retrieval of dermoscopic images, offering a time-lapse comparative analysis of the cutaneous pigmented lesions at different time intervals. The main interest of the technique relates to atypical melanocytic lesions, which are impossible to diagnose by OD as being either benign nevi or very early melanomas. Besides pathological examination, which requires prior excision and diagnoses the nature of the lesions, only the monitoring of the evolution of the lesions helps determining its benign or malignant nature. SDDI has been proven to favor earlier melanoma detection, including melanomas lacking clinical and dermoscopic features for melanoma (so-called “featureless melanomas”) [5]–[8]. The literature about SDDI often focuses on its use in patients with a large number of nevi. Nevertheless, SDDI is also commonly used to monitor a single or a small number of atypical melanocytic lesions [9], [10].

Both OD and SDDI have two main objectives: (i) to enhance sensitivity in the melanoma detection, (ii) to increase specificity through reduction of unnecessary excisions of benign lesions.

The present observational study, performed in a clinical setting, focused on patients with one-to-three atypical melanocytic lesions (i.e., lesions considered as suspicious by OD examination) and studied them from a medico-economic point of view. The objective was to compare the costs related to the process of melanoma detection in two situations: (i) the dermatologist, well trained in OD but without access to SDDI, was obliged to excise the lesions to exclude a melanoma, (ii) the dermatologist, well trained in OD and SDDI, had the choice between excision upfront or follow-up by SDDI, leading or not to subsequent excision.

## Materials and Methods

### Design overview

The present study is a cost comparison of two intervention options for patients presenting to dermatologists because of the patient’s concern for melanoma, and having one-to-three atypical/suspicious nevi. The two options were: (i) excision of all suspicious lesions and (ii) excision of highly suspicious and SDDI of slightly or moderately suspicious lesions. It is an observational study of the two options in terms of costs with a common clinical outcome (excisions of benign lesions per patient). We use the observations in a decision tree model to make inferences on the incremental costs between the two patient groups (on a per-patient basis through bootstrapping).

We used the database of the DEPIMELA observational study presented elsewhere [11]. In brief, the inclusion period of the DEPIMELA study ran from 1/10/2009 to 30/9/2010. The present study included all the consecutive patients with one-to-three atypical melanocytic lesions seen during the DEPIMELA study by (i) dermatologists who used OD with adequate training (Group 1) and (ii) dermatologists who used OD and who, in addition, had access to SDDI, with adequate training in both techniques (Group 2). Patients with more than three atypical nevi were excluded from this study. Patients who were already monitored by SDDI for atypical nevi were also excluded. The lesions monitored by SDDI were selected atypical melanocytic lesions. The dermatologists having access to SDDI used the latter only if they considered it helpful in addition to OD (i.e., for difficult lesions). This is referred to as “selective use of SDDI”.

The main aim of this study was to compare the costs related to the process of melanoma in Groups 1 and 2. We excluded the costs of melanoma excisions and pathology because these should be the same for each correctly diagnosed melanoma, irrespective of which group they belonged to. Because of this exclusion, the costs were referred as “extra-costs” in this paper. In Group 1, the extra-costs included costs of excision and pathology of benign lesions excised. In Group 2, the extra-costs included costs of SDDI and/or costs of excision and pathology of benign lesions excised.

OD was performed with a Delta 20 Dermoscope (Heine, Herrshing, Germany) or a Dermoscope DermoGenius Basic II (Linos Photonics, Munich, Germany). For SDDI, the dermatologists in our study used the FotoFinder Dermoscope (Teachscreen Software, Bad Birnbach, Germany).

Reference diagnosis was pathological analysis in Group 1, pathological analysis or stability of the SDDI picture after a minimum of three months in Group 2.

The DEPIMELA study was approved on 28 November 2008 by the ethics committee of the Université catholique de Louvain (number B40320085012). Part of the medical file was copied by the dermatologist in a structured document to provide the elements needed by the study. At that time, the patient gave his verbal consent, which was acknowledged by the dermatologist. A written consent was not required in this kind of observational study. Indeed, the subject of the present survey was the practice of the dermatologists and the economical consequences of the technique they used, rather than the patients themselves. The latter freely chose their dermatologist irrespective of the study (and were not randomized). The ethics committee approved this procedure.

### Setting and participants

#### Group 1

Twelve volunteer dermatologists adequately trained in OD were recruited from French-speaking Belgian private and hospital practices without access to SDDI. Populations seen by these dermatologists were not statistically different in terms of age, sex and risk factors for melanoma (p values>0.1). We included the following risk factors: (i) a personal history of melanoma, (ii) a family history of melanoma (at least 2 melanomas in first-degree relatives), (iii) Fitzpatrick’s skin phototype I (very fair skin) or (iv) a stay of at least one year in a tropical country before the age of 15. They were a priori not different in terms of social status and access to a dermatologist (approximation assessed according to the fact that all the dermatologists had mixed private/non private facilities). The dermatologists were considered as adequately trained if (i) they had received more than ten hours of initial training in OD and (ii) they maintained self-training. They included all consecutive patients who had one-to-three melanocytic lesions which were excised because of low to high suspicion of melanoma. Patients who had asked for the excision for cosmetic or comfort reasons were excluded, if the dermatologist had no doubt about the benignancy of the lesion.

#### Group 2

Ten dermatologists from an academic dermatology department (Cliniques Universitaires St Luc, Brussels, Belgium), agreed to refer to the department’s pigmented lesion clinic (PLC) all their patients with any melanoma suspicion and any lesion they would have removed to exclude a melanoma. The PLC was run by two dermatologists adequately trained in OD and SDDI. The PLC dermatologist decided either to excise the lesions or to monitor these by SDDI. The reasons for excision were: (i) high suspicion of melanoma in a flat lesion, (ii) any suspicion of melanoma in a raised lesion (in order not to miss an advanced melanoma), and (iii) any suspicion of melanoma if the patient refused the follow-up by SDDI.

The monitoring by SDDI was conducted after three months and after nine additional months. Three months is the commonly accepted interval before the first follow-up [12] and has been shown to detect 93% of *in situ* melanomas of the non-lentigo maligna type and 96% of invasive melanomas [5]. Patients missing the first check-up were called and offered another appointment. A second follow-up after one year is ideal to detect the remaining proportion of so-called “slow-growing” melanomas [13]. This second follow-up was presented as optional for patients with moderately atypical lesions and without any of the aforementioned risk factors for melanoma. In these cases, patients were instructed to observe their lesion and return if any change was observed. Excision was performed if any significant change was observed, according to the literature [7], [12].

### Outcomes

The present study analysed a part of the DEPIMELA study database from an economic point of view. The aim was to compare the extra-costs in both Groups. In Group 1, the extra-costs included the excision costs of all the benign nevi excised because of having been considered as suspicious by dermatologists well trained in OD: in Group 2, the extra-costs included the SDDI follow-up costs and/or benign lesions excision costs when patients were monitored in a place where SDDI was available. We first computed the observed costs in both groups, then the simulated costs of both groups obtained from a decision-tree model. The latter allowed us to obtain an idea of the uncertainty of our results.

### Costs

Unit costs were based on official tariffs and charges in Belgium in 2012 [14] (Table 1). Although the coverage of OD and SDDI by the national Belgian health care system only became effective on 1/3/2014, we used the 2012 figures because we already knew their official reimbursement amounts in November 2012. The SDDI examination official cost includes: (a) total body examination by OD, (b) electronic storage of atypical nevi dermoscopic pictures and (c) localization of these nevi on the body. This cost is added to the cost of the consultation. Currently, the following restrictions for reimbursement of the SDDI examination costs apply in Belgium: (i) SDDI is only reimbursed for patients with a personal history of melanoma, a family history of melanoma (at least two melanomas in first-degree relatives) or patients with Atypical Mole Syndrome (AMS) (simplified from Newton Bishop’s definition: ≥100 nevi and ≥2 atypical nevi) [15]; (ii) irrespective of the number of OD and/or SDDI examinations per year, only one OD and only one SDDI examinations are reimbursed per year. Nevertheless, in our comparative cost analysis, we applied these unit costs to every unit of associated resource use, as if (i) the reimbursement would be effective for all the patients (irrespective of their history or phenotype) and (ii) the number of SDDI reimbursements in a year would not be limited.

**Table 1:** Current unit costs in Belgium (2012), expressed in Euros.

Item	Cost
Dermatologist’s consultation cost	28.88
General practitioner’s consultation cost	23.67
Cutaneous tumor excision with suture	54.10
Second cutaneous tumor excision with suture	27.05
Cutaneous tumor(s) pathology	62.02
Immunohistochemistry	25.41
Optical dermoscopy	6.39
Sequential digital dermoscopy imaging	23.22

Costs are the same in academic hospitals and non-academic hospitals or private practices.

The costs were calculated using the currently prevailing treatment pathways in Belgium. We considered the most common situation which is the following. The patient is examined during a first consultation and the possible decision to excise a lesion is discussed at this time. The excision is performed a few days or weeks thereafter. The stitches are removed and the scar is checked by the general practitioner. If several excisions are performed the same day, the second and the third excision costs are divided by two and the pathology cost remains the same for one or more lesions. We took into account the observed number of patients with two or three excisions made the same day to calculate the extra-costs. Regarding immunohistochemistry, it is generally admitted that this is useful to exclude melanomas in cases of nevi with severe atypia. These nevi are much more frequent in the second group as demonstrated in the DEPIMELA study [11]. We assumed that two immunostains (HMB 45 and a cocktail of antibodies including gp100, tyrosinase and MelanA) were performed in a number of benign nevi equivalent to the number of melanomas in each group. In accordance with Belgian guidelines [16], personal non-medical costs such as travel fare as well as indirect costs due to absence from work for the consultations or because of scarring were not taken into account.

### Classification bias

It cannot be excluded that some lesions followed by SDDI were in fact (very) slow-growing melanomas. Nevertheless, the vast majority of the patients continued to be monitored in our institution. We have, therefore, followed up these patients for more than three years. In addition, it cannot be excluded that some melanomas could have been missed in the first group but would have been correctly diagnosed if included in the second group.

### Selection bias

Social status and access to a dermatologist were not measured and can therefore be considered as a potential bias.

### Data analysis

Both groups were compared using a cost comparison focused on the costs of unnecessary excision and/or follow-up costs. These costs were defined as “extra-costs” because the costs of melanoma excisions and pathology were not taken into account (these should be the same for each correctly diagnosed melanoma, irrespective of which group they belonged to). The extra-costs were calculated by patient and not by lesion because several lesions were excised or monitored by SDDI at the same time, which reduced the costs per patient in both cases. The extra-costs were then divided by the number of melanomas detected in each group, to obtain an “additional cost per diagnosed melanoma”.

### Statistical analysis

A decision-tree model was developed to assess the statistical significance of the total extra-cost difference between both groups [17]. A probabilistic sensitivity analysis (PSA) was integrated in the model to express parameter uncertainty [18]. Data derived parameter distributions were defined based on the clinical characteristics of both groups (Table 2), and the unit costs listed in table 1 were attributed on the simulated values drawn from these parameter distributions. All calculations were performed in R 3.0.1 (R Core Team, 2013) [19]. For each of the 10,000 iterations of the model, a value was sampled from each parameter distribution, such that a distribution of 10,000 estimates was obtained from each outcome (with the cost difference between both groups being the primary outcome of interest). We will report the proportion of iterations resulting in OD being more expensive than selective SDDI. To assess which input parameters had the greatest influence on the variability in the overall output, i.e. the cost difference between both groups, we complemented the PSA with a variable importance analysis. We calculated standardized regression coefficients as a measure of variable importance. These were obtained by first standardizing the input parameters and the output (i.e. subtracting the mean and dividing by the standard deviation), and subsequently regressing the standardized input parameters against the standardized output. R code is available as supplementary file to the manuscript (Code S1).

**Table 2:** Parameter distributions for probabilistic uncertainty analysis.

Parameter	Group 1*Distribution*	Group 1*Mean*	Group 1*Range*	Group 2*Distribution*	Group 2*Mean*	Group 2*Range*
Number of patients with unnecessary excisions	Binomial(7434, 533/7434)	533	490–577	Binomial(1926, 79/1926)	79	62–96
Proportion of patients with>1 unnecessary excision	Beta (33, 500)	0.06	0.04–0.08	0	–	–
Average number of unnecessary excisions per patient	Gamma (570, 533)	1.07	0.98–1.16	1	–	–
Number of excised Melanomas	Poisson (70)	70	54–87	Poisson (32)	32	21–44
Number of patients registered by SDDI at inclusion time	–	–	–	Poisson (124)	124	103–146
Proportion of patients followed up by SDDI at 3–6 months	–	–	–	1	–	–
Proportion of patients followed up by SDDI at 12 months	–	–	–	Beta (90, 34)	0.73	0.64–0.80

SDDI = Sequential Digital Dermoscopy Imaging.

The mean is defined as the mean of the distribution. The range is constructed as the 2.5^th^ and 97.5^th^ of the concerned distribution. We assumed the different parameters in that table to be independent.

## Results

**Figure 1:**
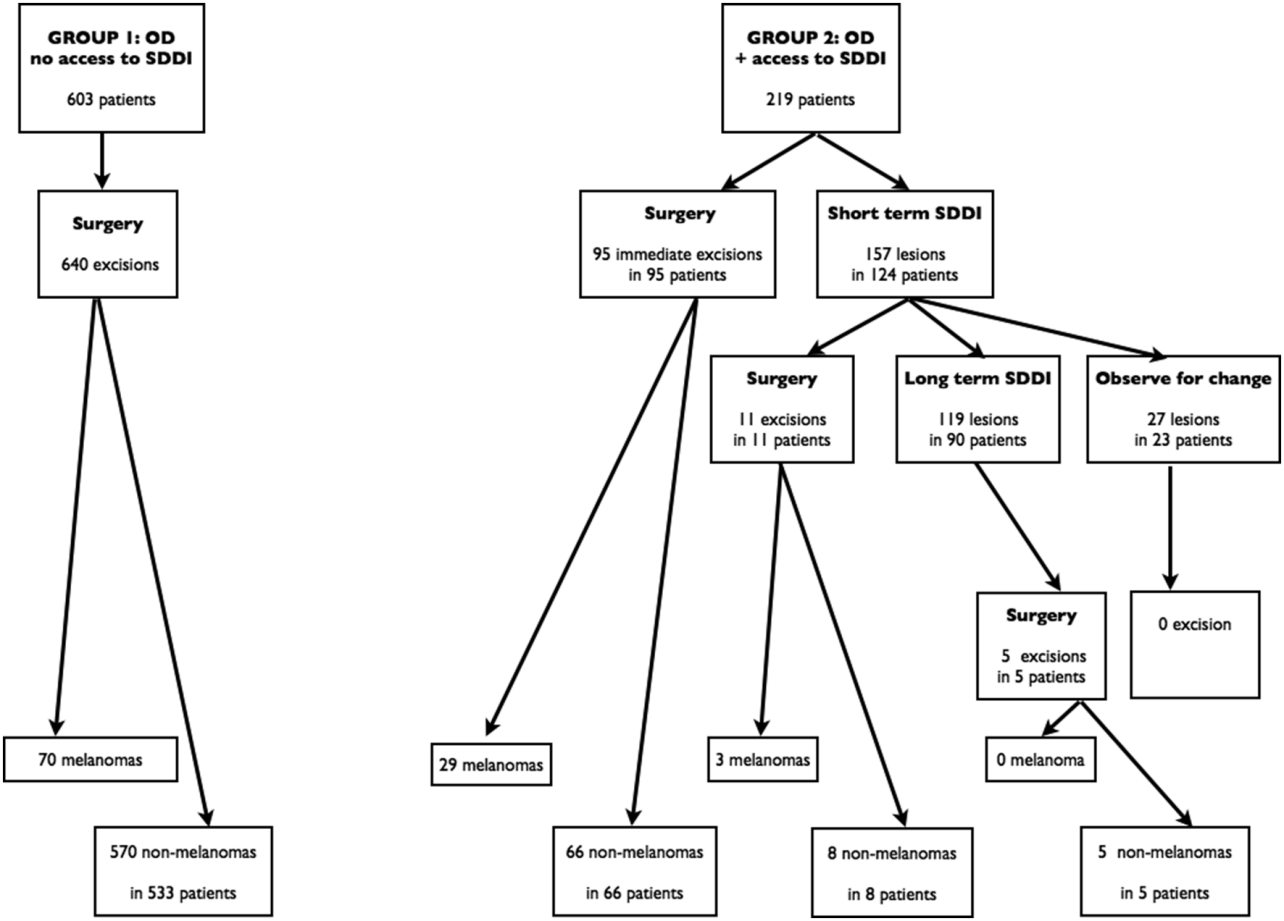
Study flowchart. The evolution of the patients is divided into two groups. In Group 1, patients were examined by dermatologists adequately trained in optical dermoscopy (OD). In Group 2, patients were examined by dermatologists adequately trained in optical dermoscopy and who had access to sequential digital dermoscopy imaging (SDDI).

Patients’ progress in Group 1 and 2 are summarized in Figure 1.

### Group 1

Of the 7,434 patients examined during one year for melanoma detection, 603 were eligible for this study and underwent surgery. There was no patient with more than three atypical/suspicious nevi who had to be excluded (probably because patients with many atypical nevi were either referred to a PLC or underwent regular excisions of their new atypical/suspicious nevi). The mean age of these 603 patients was 41 years (from 4 to 86 years) and the sex ratio (M:F) was 0.57. Six hundred and forty excisions were performed, leading to the diagnosis of 70 melanomas. The melanoma/non-melanoma ratio (M/NM-R) was 1/8.14. Regarding benign lesions, excision and pathology observed costs were €112,920, which can also be expressed as €1,613 for every melanoma excised.

### Group 2

During the same year, 1,926 patients were examined for melanoma detection and 219 were eligible for the present study. The mean age of these 219 patients was 39 years (from 14 to 88 years) and the sex ratio (M:F) was 0.58. Eighty-eight patients required immediate excision of suspicious lesions, leading to the diagnosis of 29 melanomas. Seven patients underwent excision because they refused the SDDI monitoring; all these lesions were diagnosed as benign by pathology. The 124 remaining patients agreed to be monitored by SDDI for a total of 157 lesions. The short-term follow-up was planned after three months. Six patients missed their appointment and were re-contacted. Finally, all the 124 patients were examined within six months. Eleven lesions had changed after 3 months and were excised, three of these were melanomas. A second check by SDDI after one year was suggested to the 113 remaining patients. The dermatologists insisted on the importance of this examination in patients with very atypical lesions and/or with identified other melanoma risk factors. All these patients came to this one year appointment, as well as many other patients of this group, even if this appointment had been presented to them as optional. Only 23 patients did not show-up after 18 months. They all were clearly informed they had to observe their nevi and to request a visit in case of change. However, none came back for this reason. The second follow-up visit led to the excision of five monitored lesions, which were all diagnosed as benign by pathology. Finally, 32 melanomas and 79 non-melanomas were excised: the total M/NM-R was 1/2.47. The three melanomas excised after the short-term SDDI visit were *in situ* for two of them and very early invasive (Clark level II, 0.2 mm of Breslow’s thickness) for the remaining case. The total observed extra-cost was €33,658 and was nearly equally distributed between excision and pathology costs (€17,233) and SDDI follow-up costs (€16,425). The extra-cost, for each melanoma excised, was €1,052 in Group 2. The follow-up period for the DEPIMELA study was one year. In addition, most patients have been seen in the institution during the following 2.5 years and any melanoma was reported.

### Comparison of Group 1 and Group 2

**Figure 2:**
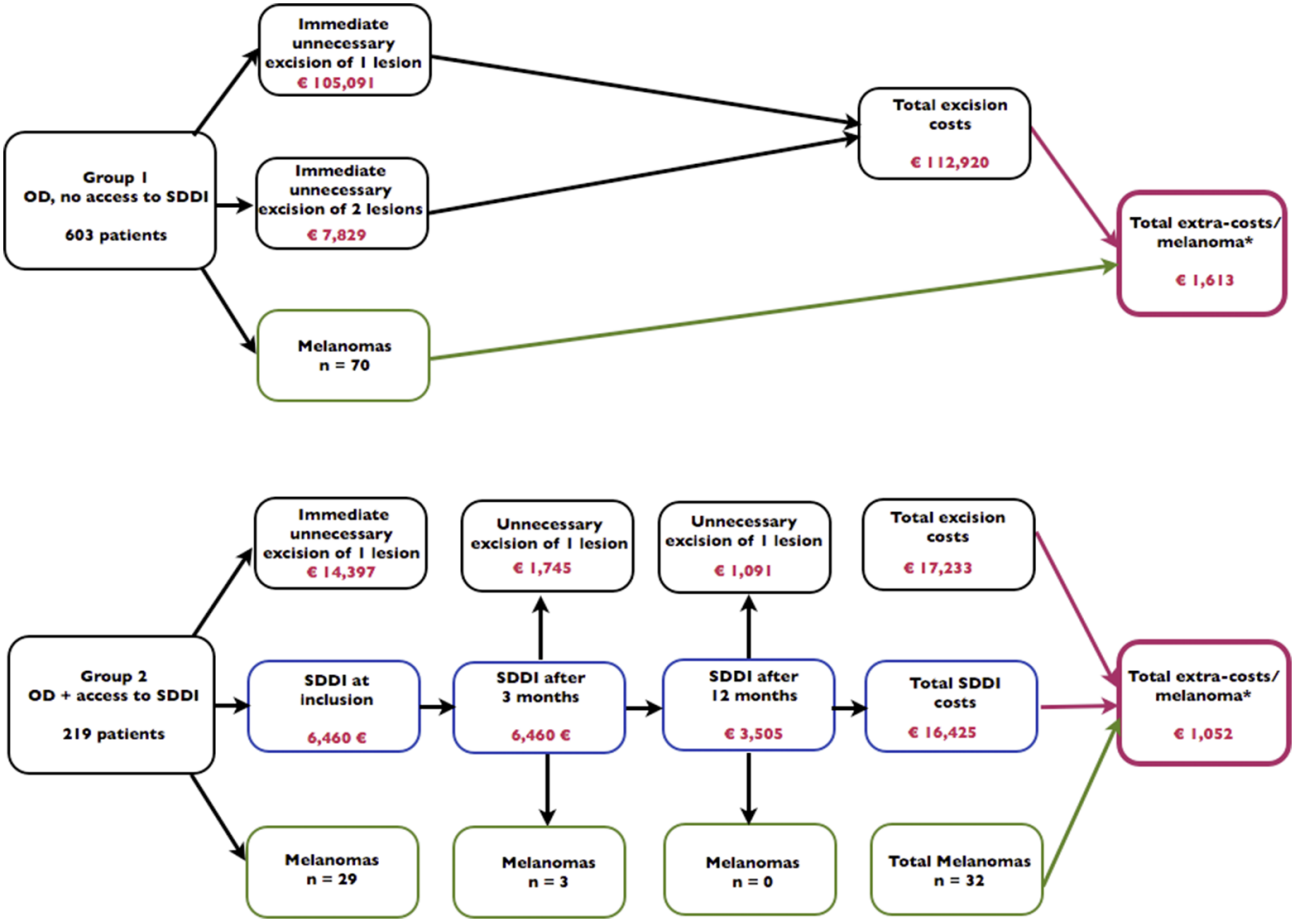
Observed total direct extra-costs distributed in the decision-tree model. This figure shows the observed total direct extra-costs distributed in a decision tree model. In Group 1, patients were examined by dermatologists adequately trained in optical dermoscopy (OD). In Group 2, patients were examined by dermatologists adequately trained in optical dermoscopy and who had access to sequential digital dermoscopy imaging (SDDI). *Melanoma excision costs are not taken into account because these should be the same for each correctly diagnosed melanoma, irrespective of which group they belonged to.

**Figure 3:**
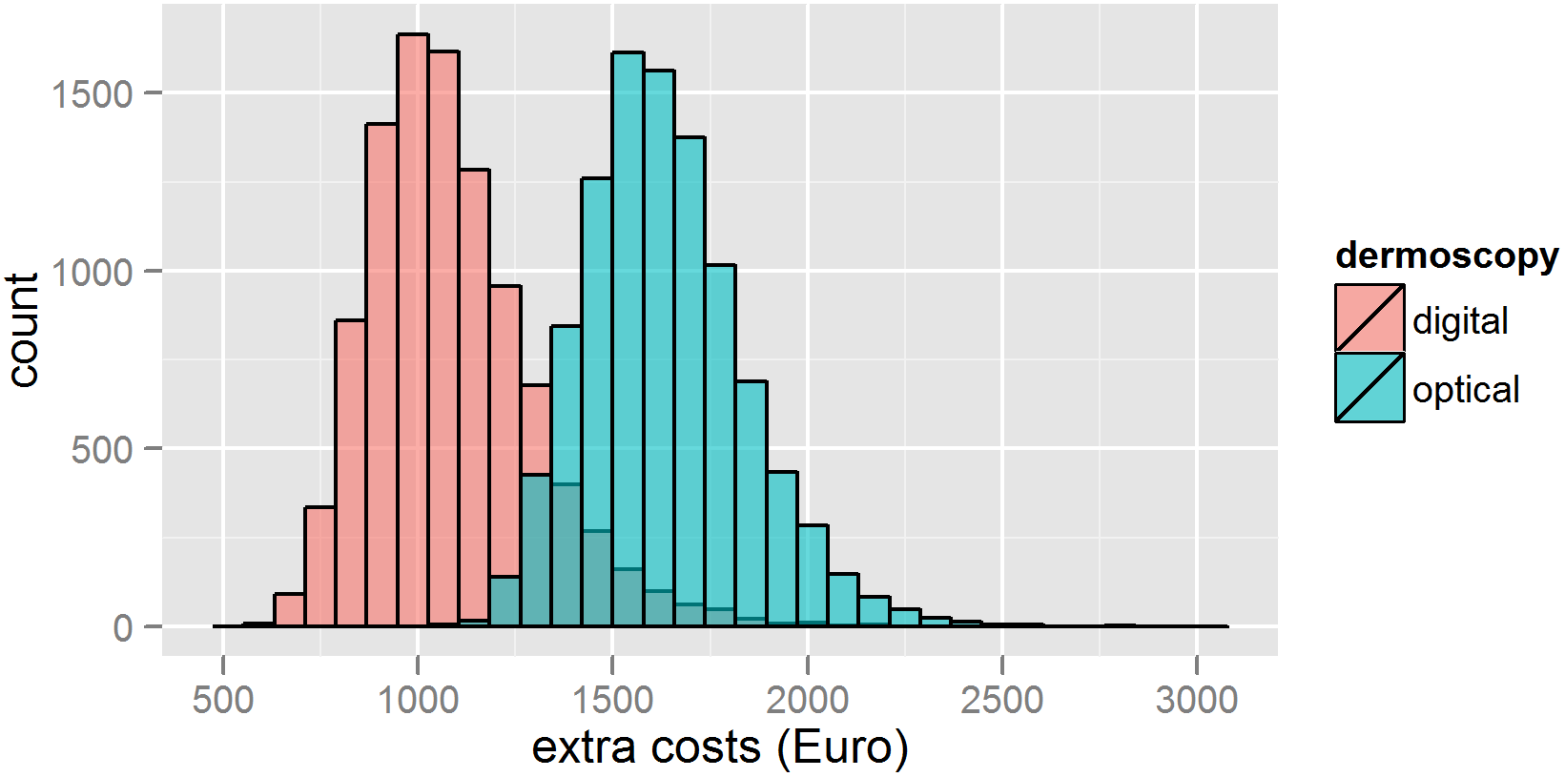
Histogram of the estimated extra costs of sequential digital dermoscopy imaging versus optical dermoscopy. The extra-costs are defined as the costs of excision and pathology of benign lesions and/or the costs of follow-up by sequential digital dermoscopy imaging. These extra-costs are divided by the number of melanomas diagnosed in each group.

**Figure 4:**
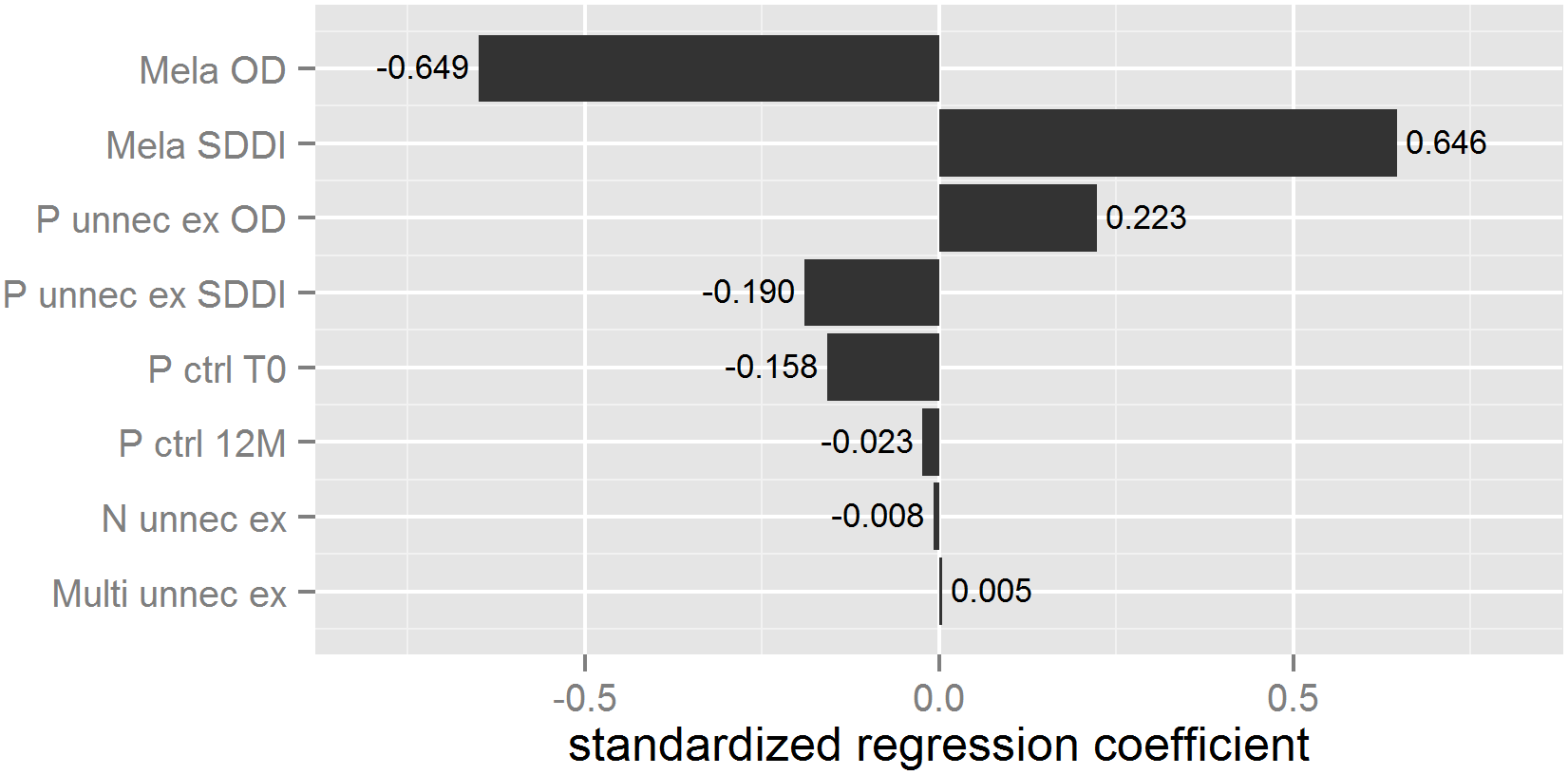
Sensitivity analysis: tornado graph. *Mela OD*: number of excised melanomas in the optical dermoscopy (OD) group; *Mela SDDI*: number of excised melanomas in the sequential digital dermoscopy imaging (SDDI) group; *P unnec ex OD*: number of patients with unnecessary excisions in OD group; *P unnec ex SDDI*: number of patients with unnecessary excisions in SDDI group; *P ctrl T0*: number of patients registered by SDDI at inclusion time; *P ctrl 12 M*: proportion of patients followed-up by SDDI at 12 months; *N unnec ex*: average number of unnecessary excisions per patient; *Multi unnec ex*: proportion of patients with >1 unnecessary excision.

The observed total direct extra-costs in Group 1 versus Group 2 are shown Figure 2. This figure shows also the decision-tree model which was used to simulate the extra-costs resulting from melanoma detection. Parameter distributions for probabilistic uncertainty analysis are presented in Table 2. Through this probabilistic uncertainty analysis we estimated the mean cost difference between Group 2 and Group 1 at €548 (95% credibility interval: 65–1856) (Figure 3, Table 3). The extra-costs per melanoma excised presented in Table 3 (€1,633 in Group 1 and €1,085 in Group 2) and the extra-cost per melanoma excised presented in Figure 2 (€1,613 in Group 1 and €1,052 in Group 2) are different because the first ones were simulated and the second ones were observed. The proportion of iterations, resulting in OD being more expensive than selective SDDI, was equal to 96.5%. At a 5% significance level, this indicates a significant statistical difference between the extra-costs in both groups. Figure 4 shows the tornado graph (i.e., standardized regression coefficients of the different parameters, ranked according to their absolute values). The coefficients reflect how many standard deviations the output will change per standard deviation increase in an input. The largest source of uncertainty is, logically, the number of excised melanomas in both groups (denoted “Mela OD” and “Mela SDDI”). The R-squared of the variable importance regression model was 0.95.

**Table 3:** Simulated costs of excision, pathology and/or follow-up of benign lesions, expressed in Euros (mean+95% credibility interval).

	Group 1^a^	Group 2^a^	Difference^b^
Excisions*	76,536 (70,314–82,850)	10,710 (8403–13,011)	65,82 (60,289–78,320)
Pathology**	36,394 (33,512–39,355)	6529 (5240–7893)	29,86 (27,160–35,586)
Follow-up by SDDI***	-	16,419 (13,510–19,475)	−16,419 (−18,967– −10,971)
Total	112,929 (103,947–121,967)	33,658 (29,052–38,257)	79,271 (70,902–98,963)
Total/melanoma diagnosed	1633 (1289–2091)	1085 (758–1588)	548 (65–1856)

*Includes consultations, optical dermoscopy in Group 1, and surgery.

**Includes classical pathology and immunohistochemistry for very atypical nevi.

***Includes consultations and sequential digital dermoscopy imaging.

a Two-sided 95% credibility interval (constructed as the distribution’s 2.5^th^ and 97.5^th^ percentile).

b One-sided upper 95% credibility interval (constructed as the distribution’s 5^th^ and 100^th^ percentile).

## Discussion

The present observational study, performed in a clinical setting, focused on patients with one-to-three suspicious melanocytic lesions (i.e., lesions considered as suspicious by OD examination) and studied them from a medico-economic point of view. From a sample of 822 patients, we showed that, when a dermatologist well trained in dermoscopy had the choice between excision and follow-up by SDDI (leading or not to subsequent excision), the extra-costs were statistically lower than if he was obliged to excise the lesions in order to rule out melanoma. Extra-costs included costs of SDDI and/or costs of excision and pathology of benign lesions excised.

To the best of our knowledge, this is the first medico-economic study on SDDI. This technique is reimbursed in Belgium and in several other European countries but, in most countries, only for patients at high risk for melanoma (i.e., mainly patients with personal history of melanoma and/or AMS). It has probably been assumed that SDDI is cost-effective in these groups. We studied patients with one-to-three suspicious melanocytic lesions. Most of them were at low risk for melanoma. In the Belgian context, the costs borne by these patients are higher with selective SDDI follow-up (where reimbursement is not covered) than in the case of systematic excision (which is completely reimbursed). However, we have shown that the costs borne by the national health care system were higher in the case of systematic excision, including if the SDDI costs are reimbursed without restriction. As a result, we believe that, in Belgium, all patients’ SDDI costs should be reimbursed.

We excluded patients with many atypical nevi because most of them are classified in the AMS [15]. This syndrome increases the melanoma risk, not only because a melanoma can arise on an atypical nevus but, predominantly, because the skin of these patients is at high risk of generating melanomas from isolated melanocytes. Therefore, these patients must, ideally, be monitored by SDDI for many years and the method of analyzing the costs of this follow-up should be different from the method used in the present study.

The present study focused on patients with one-to-three suspicious melanocytic lesions, divided into two groups. In Group 1, patients were examined by dermatologists adequately trained in OD. In Group 2, patients were examined by dermatologists adequately trained in OD and having access to SDDI if needed. The extra-costs in the first and in the second group were respectively €1,613 and €1,052 per melanoma diagnosed. The gain linked to SDDI selective use was more than €500 per melanoma diagnosed. Since the acquisition cost of the SDDI equipment was around €20,000 in 2012, its appropriate use in hospitals could win back the excess investment for the health care payer to correctly diagnose melanoma (even when ignoring SDDI’s benefits to AMS patients, as we did here). Nevertheless, irrefutable evidence of this would require specific prospective studies.

The cost difference between both groups is closely related to the M/NM-Rs differences. The M/NM-Rs were 1/8.14 in Group 1 and 1/2.47 in Group 2. It could be argued that this difference between both M/NM-Rs was linked to a higher level of experience in OD in Group 2. However, the skill level in OD is limited by the technology itself. Most atypical nevi cannot be differentiated from very early melanomas and their evolution over time is the only way to have more information. It could also be argued that this difference was partially due to a different population in a University hospital (Group 2). Interestingly, if all the lesions considered as moderately suspicious and monitored by SDDI without final excision had been added to the non-melanoma lesions excised in Group 2, the M/NM-R in Group 2 would be 1/7.78. This number is not statistically different from the 1/8.14 in Group 1 (p value = 0.2).

Even with the availability of SDDI, the number of excised benign lesions will never be reduced to zero for several reasons: (i) raised ambiguous melanocytic lesions should not be monitored because of the risk of missing an advanced melanoma, (ii) excision of Spitz nevi is generally recommended, even if it is controversial for typical lesions in children [20], (iii) some clinically and dermoscopically atypical nevi mimic melanomas and will always be excised.

Kittler et al. found in an experimental study that the possibility of follow-up by SDDI reduced the number of benign excised lesions, but only by very experienced dermoscopists [21]. This allows us to insist on the fact that our results cannot be extrapolated to beginners in dermoscopy: if the excision rate does not decrease thanks to SDDI availability, the costs would probably be higher in the second group. As mentioned by Kittler et al in the same publication [21], compliance is a critical point for the success of monitoring. Our rate of compliance was very good, compared to other studies [5], [22]. Only 3.6% of patients had to be re-contacted.

This study is not a cost-effectiveness analysis, because only costs were compared between both groups. A further comparison based on cost-effectiveness would require relating cost-differences between the groups to effect-differences. In economic evaluation, these effect-differences typically include improvements in health related quality of life (HRQoL) and survival. HRQoL was expected to be higher in Group 2, because unnecessary surgeries and scars were avoided. Concerning the survival, the three melanomas found by SDDI were *in situ* or very early invasive (Clark level II, Breslow’s thickness 0.2 mm). *In situ* melanoma never metastasize. The 10–20 year melanoma specific survival rates for melanomas with Clark II level and Breslow thickness <0.25 have been reported to be close to 100% (98.3 to 100%) [23], [24]. The other melanoma patients, diagnosed with OD in both groups, had a life expectancy not linked to the group they belonged to, but only linked to the stage of their melanoma.

Our analysis provided clear indications that the average per-patient costs were lower in Group 2 than in Group 1. Although no statistical testing has been undertaken in the absence of prospective survival studies, the current consensus in the literature is that, on average, survival is expected to be similar in both groups [23], [24]. Furthermore, it seems intuitively highly likely that the HRQoL per average patient was higher in Group 2 than in Group 1, for the reasons we explained above. Therefore, with lower costs, similar survival, and higher HRQoL, Group 2 was likely to dominate Group 1. However, since there is no separate study showing significant effectiveness and since costs and effects are likely to be correlated, a formal economic evaluation alongside a prospective clinical trial would be required to provide irrefutable evidence on this matter [25]. Perhaps our study can serve as a further incentive to set up such a (albeit costly) study. Fundamentally our study is limited by the relatively small sample of patients diagnosed by SDDI plus the lack of any study comparing OD *versus* OD and selective use of SDDI in terms of effectiveness in the general population, let alone in terms of cost-effectiveness.

Regarding SDDI literature, on the one hand, a meta-analysis of 14 SDDI studies concluded on the safety of the technique that among 383 melanomas detected, more than half were *in situ* and the rest were early invasive melanomas (thickness always thinner than 1 mm) [26]. All these melanomas had an excellent prognosis but the thickest ones had a low risk of metastasis. It is, however, unclear whether the slightly later detection of the melanomas excised because of a change on SDDI pictures would have had an impact on melanoma-related mortality. On the other hand, more than half of melanomas detected by SDDI are “featureless melanomas”, i.e. melanomas which would perhaps have been missed by OD [7]. Finally, the benefit of very early detection should be weighed against the risk of significant progression of monitored melanomas, which is probably very low but not equal to zero. Nevertheless, regarding the SDDI use in a very large number of PLC in the world, we can conclude that experts in dermoscopy assume SDDI to be safe (when performed by experienced dermoscopists in compliant patients).

Our results can only partially be extrapolated to other countries where SDDI is not reimbursed or where the price and/or the conditions of reimbursement are very different from those chosen in this study. The price of consultations, excisions and pathology vary also from one country to another. Our results should be confirmed by a multicenter randomized study and could be extrapolated to other countries if the same calculations are made using the specific prices of each country.

Our study has some limitations. The excision and pathology of benign lesions are probably not always useless: some very dysplastic nevi and some atypical Spitz nevi, despite a final diagnosis of benignancy, will be treated as melanomas because the pathologist cannot completely exclude a melanoma diagnosis [20], [27]. Nevertheless, these cases are rare. This is an observational study and therefore patients were not randomized between the two groups. The potential bias are the following. First, it cannot be excluded that some lesions from Group 2, considered as benign after a three or twelve month follow-up, were slow-growing melanomas and had been lost from our follow-up. The subgroup of so-called “slow-growing melanomas” is diagnosed by SDDI after a median period of 20 months [13]. The prevalence of such melanomas is unknown but seems to be low. Even if very rare cases are diagnosed by SDDI after more than five years, the risk of having missed such melanomas in our study is extremely low. Return to the PLC if any change was observed could be burdensome to patients. If only one melanoma was missed, we must consider two extreme situations. If the melanoma was diagnosed in a very early stage (*in situ* or Clark II), our conclusions are unchanged because the extra-costs per melanoma in Group 2 would be lower. If the melanoma was diagnosed in an advanced stage, the use of SDDI must then be considered as unsafe. Second, it is possible that some featureless melanomas were missed in Group 1 and would have been diagnosed by SDDI. Although SDDI permits an earlier diagnosis due to the identification of the so-called “featureless melanomas” [5]–[8], melanoma prevalence was probably similar in both groups. In terms of costs, this difference should not influence the detection costs, but perhaps the treatment costs. Third, according to some authors, part of the *in situ* melanomas would be indolent forms without metastatic potential [28]. The proportion of *in situ*/invasive melanomas is not statistically different in both groups (p value>0,1). Nevertheless, as this proportion is high (around 1/1.7), if we had to remove all the *in situ* melanomas from our study, our sample sizes would become smaller and our results may therefore no longer be statistically significant. Fourth, reimbursement driven unit costs from home to hospital were not taken into account. This could perhaps have increased the extra-costs in Group 2. Although reimbursement driven unit costs are generally low in Belgium, this might not be the case in countries with a lower population density and/or a lower dermatologist to patient ratio. Fifth, social status and access to a dermatologist were not measured and can therefore be considered as a potential bias.

## Conclusion

Assuming that SDDI would be reimbursed and easily available to all patients with atypical nevi, the present observational study showed that selective SDDI reduces the extra-costs in the process of melanoma detection in patients with one-to-three atypical nevi. It would be interesting to confirm our results, obtained from a non-randomized observational study, by a multicenter randomized study. When practiced by dermoscopy experts, SDDI allows the follow-up of benign atypical lesions, avoiding their systematic excision. The extra-costs, mostly linked to the excision of atypical nevi mimicking early melanomas, will never be reduced to zero, but could be significantly reduced by SDDI in certain conditions, as was described above.

## Supporting Information

Code S1 R code for the cost-minimization model and sensitivity analysis.
(TXT)
